# Explainable identification and mapping of trees using UAV RGB image and deep learning

**DOI:** 10.1038/s41598-020-79653-9

**Published:** 2021-01-13

**Authors:** Masanori Onishi, Takeshi Ise

**Affiliations:** 1 Graduate School of Agriculture, Kyoto University, Kyoto, Japan; 2 Field Science Education and Research Centre, Kyoto University, Kyoto, Japan

**Keywords:** Biodiversity, Conservation biology, Forestry

## Abstract

The identification and mapping of trees via remotely sensed data for application in forest management is an active area of research. Previously proposed methods using airborne and hyperspectral sensors can identify tree species with high accuracy but are costly and are thus unsuitable for small-scale forest managers. In this work, we constructed a machine vision system for tree identification and mapping using Red–Green–Blue (RGB) image taken by an unmanned aerial vehicle (UAV) and a convolutional neural network (CNN). In this system, we first calculated the slope from the three-dimensional model obtained by the UAV, and segmented the UAV RGB photograph of the forest into several tree crown objects automatically using colour and three-dimensional information and the slope model, and lastly applied object-based CNN classification for each crown image. This system succeeded in classifying seven tree classes, including several tree species with more than 90% accuracy. The guided gradient-weighted class activation mapping (Guided Grad-CAM) showed that the CNN classified trees according to their shapes and leaf contrasts, which enhances the potential of the system for classifying individual trees with similar colours in a cost-effective manner—a useful feature for forest management.

## Introduction

The accurate characterisation of tree species distribution in forest areas is an important task for forest management and forest research. In particular, management and protection of native vegetation^1^, monitoring of invasive species^2^, wildlife habitat mapping^3^, and sustainable forest management^4^ are some of the objectives of studies that require tree species distribution characterisation on a wide scale. To this end, many studies have been conducted using remote sensing data^5^. Thus far, remote sensing research with these objectives has mainly employed satellites or aircraft. In the past, much attention has been devoted to multispectral Landsat satellites, which, because of their low cost, facilitate the mapping of forest types^6^. The advantage of Landsat satellite is that it enables the coverage of vast areas, i.e. on a country scale. However, its resolution is 30 m, which does not allow easy identification of tree species. Since 2000, many studies have used very high-resolution data for tree species classification from commercial satellites, e.g. from World-view2 and QuickBird, with resolutions of 0.5 m/2.0 m and 0.6 m/2.4 m for panchromatic/multispectral data, respectively^7–10^. Further, in recent years, studies using aircraft have succeeded in identifying several tree species. The spatial resolution of images used therein is also very high: approximately 0.2–3.0 m. Most of these studies used specialised hardware such as multispectral, hyperspectral, and LiDAR sensors, and achieved high performance in identifying tree species. For example, Shen and Cao^11^ succeeded in identifying five tree species with more than 85% accuracy. Dalponte et al.^12^ identified seven tree species and no-forest class at approximately 80% accuracy. These two studies revealed that combining hyperspectral sensor and LiDAR data acquired by an airplane is superior to the use of only multispectral or hyperspectral sensor. However, although this combination of data acquisition methods performs superiorly, the equipment involved therein is highly expensive. These approaches utilising multi/hyperspectral data have often experienced problems. One of these involves the spectral features, which can differ not only between species, but also across densities of leaves, health conditions, and background noises such as understory vegetation or bare soil^1,10,13^. When there is a shadow, the spectrum of the shadow differs from that of the no-shadow area, resulting in a lower accuracy^11^. In identifying a mixed forest, the performance might be lower because multiple species are included in one pixel^9^. These challenges can be attributed to the dependence of these approaches on the use of spectral information.

In recent decades, unmanned aerial vehicles (UAVs) have been used experimentally in forestry applications^14–18^. Compared to manned aircraft, UAVs are easy-to-use, low-cost tool for remote sensing of forests. Regarding tree identification, the most important difference between manned aircraft and UAVs is that UAVs can fly near canopies and acquire extremely high-resolution images; the images from UAVs have a spatial resolution of a few centimetres. In some cases, even the tree features at the leaf level can be seen. If we can use these features in tree identification systems, it is possible to map various tree species with the use of simple red–green–blue (RGB) digital images; such a method would facilitate cost-effective monitoring with broad application potential.

Meanwhile, deep learning has become an effective tool for object detection. In recent years, studies combining deep learning and remote sensing data such as airborne LiDAR or aerial image, have exhibited high potential for individual tree detection, dead forest cover mapping, and forest damage assessment^19–22^. Among deep learning techniques, convolutional neural networks (CNNs) have demonstrated high classification performance for digital images in the computer vision field^23–25^. Thus far, other machine learning approaches such as support vector machines (SVMs) or the random forest have been used in this field. Especially SVMs, which enable us to conduct supervised non-parametric prediction, are one of the conventional machine learning methods and have been used for tree species identification^5,12,26–28^. One of the notable advantages of deep learning compared to such machine learning methods is that deep learning does not require manual feature extraction. Other machine learning methods required researchers to manually extract features: in this field, texture features and vegetation indices as well as raw band values are extracted in the process of feature extraction. As for texture features, in addition to the mean and variation values of each band, the grey-level co-occurrence matrix (GLCM) has been widely used to provide texture features for improving performance^26,27,29,30^. After the feature extractions, past studies utilising hyperspectral data sometimes conducted feature selection for reducing dimension in order to avoid the curse of dimensionality and high computational cost^11,28^. Alternatively, deep learning can utilise full feature information, especially that pertaining to the spatial relationship of pixels, which provides information regarding the textures and shapes of trees. Therefore, even when simple digital images are used, deep learning is expected to succeed in identifying trees with high detail and accuracy.

For interpreting the deep learning classification, algorithms which visualize some features of CNN’s models have been developed. Guided gradient-weighted class activation mapping (Grad-CAM) is one of such algorithms, and it can highlight particular image regions which provide meaningful information for model prediction^31^. Using this algorithm helps us to know the features that deep learning used, which means we can know whether deep learning really used detailed features such as textures of leaves and tree shapes, and it can provide understandable visual information about model performance.

In this work, we applied deep learning to RGB images taken by a UAV. This combination is expected to have a high potential for identifying tree types and tree species, even if the RGB image is acquired with a consumer-grade digital camera. Some recent studies have shown the potential for individual tree detection and classification of one or a few specific tree species^32–35^. The object detection method enables us to know the existence of the object tree species; thus, it may be suitable for finding specific tree species such as invasive tree species. On the other hand, forest mapping, which enables us to know the crown ratio to the area of each tree class, as well as existence of trees, helps us to monitor the change of the forest dynamics and predict biomass. A forest mapping system using UAVs and digital images would be a particularly cost-effective and useful tool for forest management applications.

The contributions of this study are threefold: (1) We propose a forest mapping system using UAVs and CNN classification. (2) We reveal its classification potential for several tree classes including tree types (deciduous and coniferous) and species in two seasons. To evaluate the performance of the CNN, we compared it with that of another machine learning method. In this study, we employed SVM as a machine learning platform, and the pixel values of each band and GLCM texture values as the features for machine learning. (3) We reveal what kind of tree features a CNN uses and show its importance (contribution) for classification by comparing it to other machine learning methods.

## Material and methods

### Study site

The study site was the Kamigamo Experimental Station of Kyoto University, located in a suburban area of Kyoto, Japan (Supplementary Figure S1). This area is located in a warm and humid climate zone, with an elevation of 109–225 m above sea level. The mean annual precipitation and temperature are 1582 mm and 14.6 °C, respectively. The overall area is 46.8 ha. 65% of the area is naturally generated forest, primarily consisting of Japanese cypress (*Chamaecyparis* *obtuse)* and some broad-leaved trees such as oak (*Quercus* *serrata* or *Quercus* *glauca).* Within this area, 28% is planted forest, mainly consisting of foreign coniferous species. 7% consists of sample gardens, nurseries, or buildings.

In this work, we focused on the northern part (an area of 11 ha) of the Kamigamo Experimental Station, containing a naturally regenerated forest of Japanese cypress, and a managed forest of Metasequoia (*Metasequoia* *glyptostroboides*), strobe pine (*Pinus* *strobus)*, slash pine (*Pinus* *elliottii),* and taeda pine *(Pinus* *taeda)*.

### Remote sensing data

Flight campaigns were conducted around noon in two seasons: on October 2, 2016, which is the end of the leaf season, and November 20, 2016, the peak of the fall leaf offset season. We used UAV DJI Phantom 4 (DJI, Shenzhen China). The UAV had an onboard camera with a 1/2.3 CMOS sensor that can capture RGB spectral information. The UAV was operated automatically using the DroneDeploy v2.66 application (https://ww.dronedeploy.com, Infatics Inc., San Francisco, United States). On October 2, we set flight parameters as follows: both the overlap and sidelap were set to 75%, and the flight height was set to 80 m from the take-off ground level. However, we failed to align some parts of the images; thus, we changed the overlap and height parameters to 80% and 100 m on November 20. We used 10 ground-control points (GCPs) for reducing the error of the GPS with the images. From the images taken by the UAV, we produced an orthomosaic photo and a digital surface model (DSM) using the Agisoft PhotoScan Professional v1.3.4 software (https://www.agisoft.com, Agisoft LLC, St. Petersburg, Russia). An orthomosaic photo is an image that is composed of multiple overhead images corrected for perspective and scale. The parameter settings used in generating the orthomosaic photo are shown in Supplementary Table S1. These parameters are for November 20. The parameters for October 2 differ only in that the ground sampling distance (GSD) were approximately one centimetre. GSD of the orthomosaic photo and DSM was approximately 5 cm and 10 cm, respectively.

### Segmentation and preparation of supervised data

**Figure 1:**
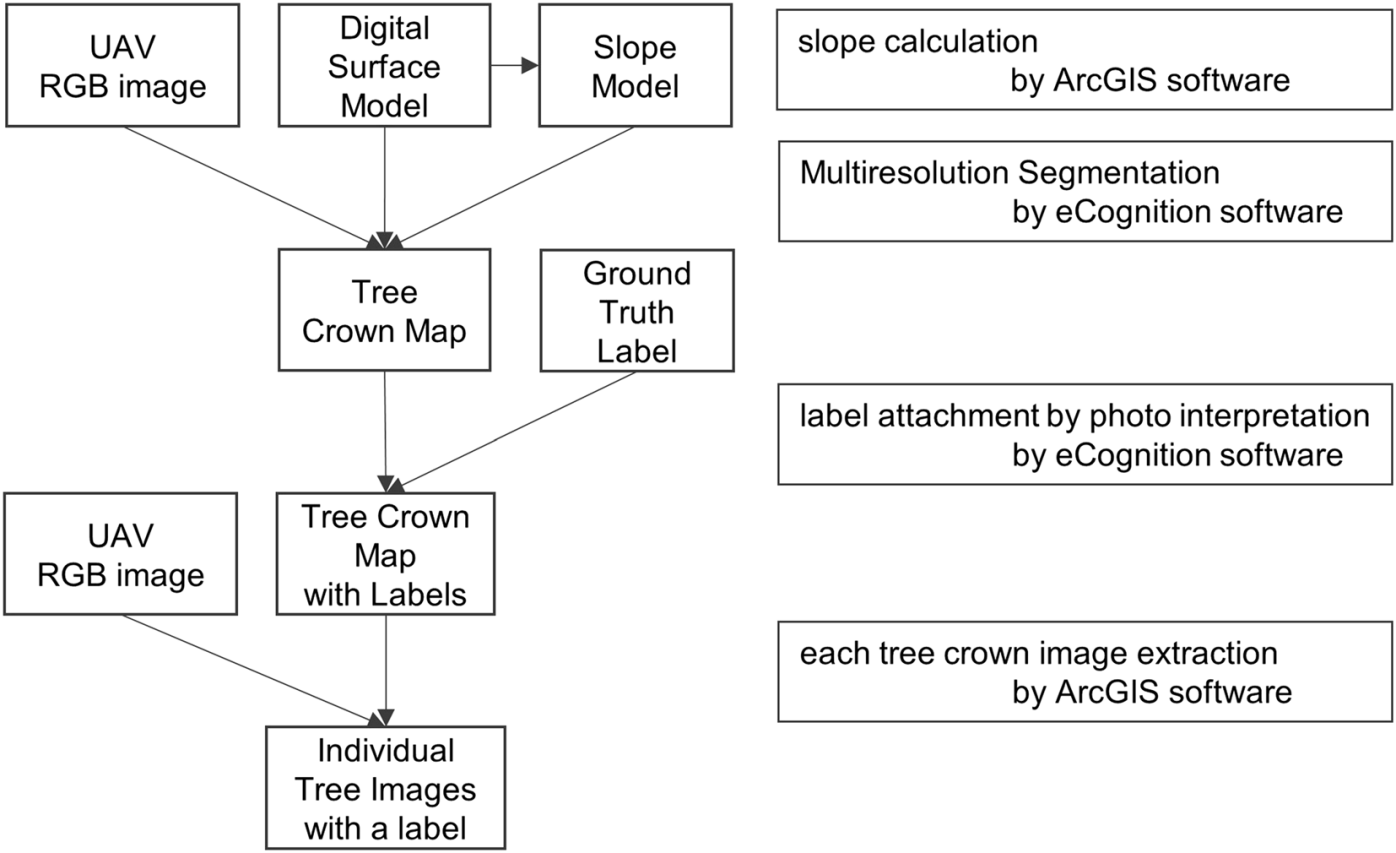
Workflow for supervised images extraction.

The technological workflow of the individual tree image segmentation and extraction method we used is summarised in Fig. 1. First, we segmented each tree crown using UAV image (orthomosaic photo), a DSM, and a slope model. Second, we visually constructed the ground truth map. Third, we extracted each tree image with a ground truth label. Further details are discussed in sections from “Object-based tree crown segmentation” to “Tree image extraction with ground truth label”.

### Object-based tree crown segmentation

**Figure 2:**
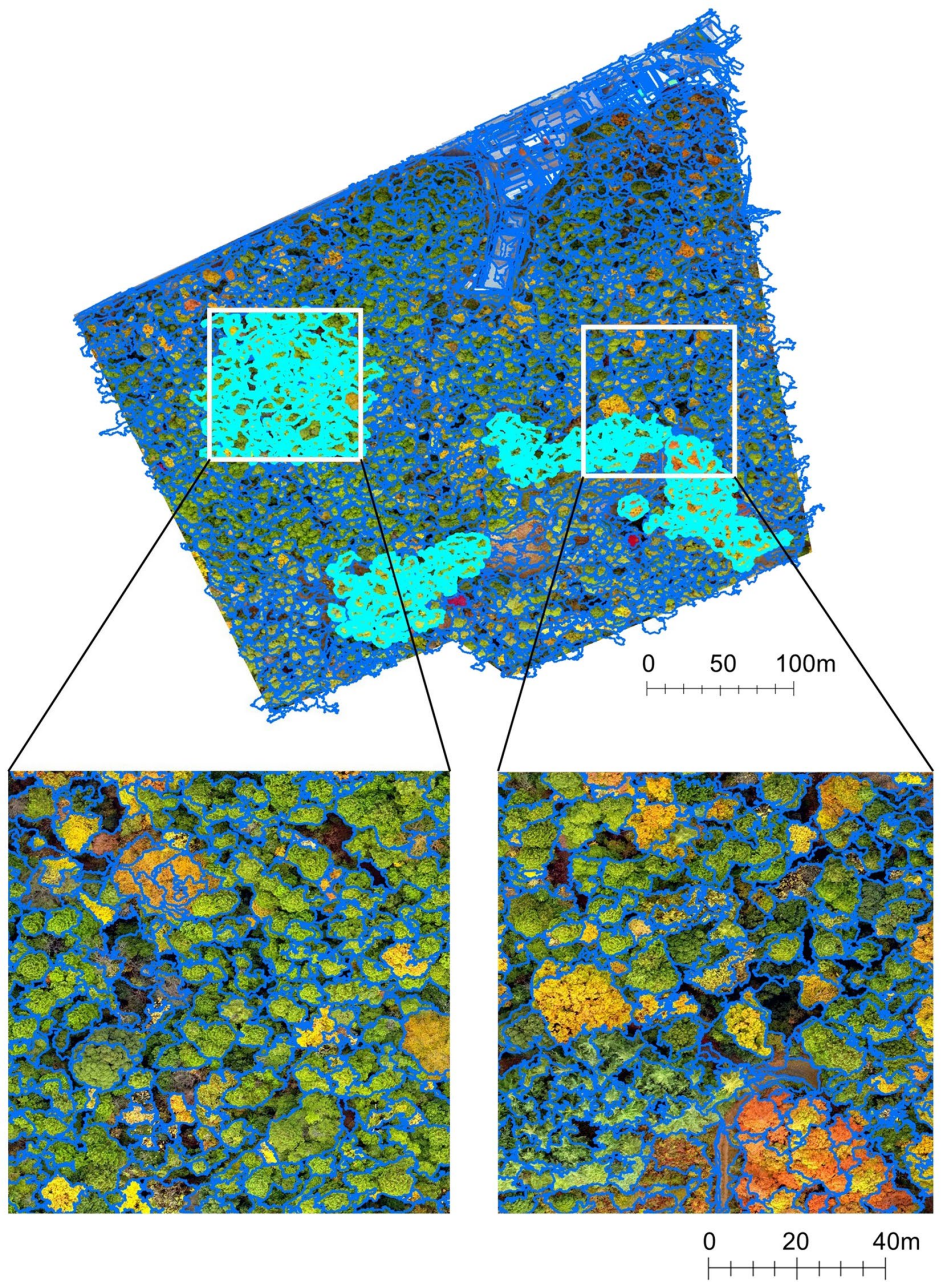
Whole area and representative enlarged tree crown map. The blue line show grid-line of segmented polygons. The white rectangle shows the location of enlarged area, and light blue polygons are used for evaluating the accuracy of tree segmentation. This map was constructed via multiresolution segmentation using colour, DSM, and Slope model. This figure was created using ArcGIS Desktop v10.6 software (https://www.esri.com, Environmental Systems Research Institute, Inc., Redlands, United States).

At the segmentation stage, we segmented at the tree level. First, we constructed a slope model by calculating the slope from the DSM using the ArcGIS Desktop v10.4 software (https://www.esri.com, Environmental Systems Research Institute, Inc., Redlands, United States). The slope model showed the maximum rate of elevation change between each cell and its neighbours, such that the borders of trees were emphasised. From the orthomosaic photo, the DSM, and the slope model, tree crown segmentation was performed in the eCognition Developer v9.0.0 software (https://www.trimble.com, Trimble, Inc., Sunnyvale, United States) using the ‘Multiresolution Segmentation’ algorithm^36^. The parameter values were adjusted by trial and error. The tree crown map made by this segmentation process is shown in Fig. 2 with enlarged images for visual confirmation of the result, and the best parameters are presented in Supplementary Table S2.

Herein, we evaluated the accuracy of the segmentation. The segmented crowns were placed into the following five categories according to their spatial relationships with the visually confirmed reference crown. The five categories, set based on a previous study ^37^, and illustrated in Supplementary Figure S2, are as follows.

(a) Matched: If the overlap of the segmented polygon and the reference crown was more than 80%, the segmented polygon was categorized as “Matched”.

(b) Nearly matched: If the overlap of the segmented polygon and the reference crown was 60–80%, the segmented polygon was categorized as “Nearly matched”.

(c) Split: If the overlap of the segmented polygon and the reference crown was 20–60%, the segmented polygon was categorized as “Split”.

(d) Merged: If multiple reference crowns covered by the segmented polygon, and even one overlap was more than 20%, the segmented polygon was categorized as “Merged”. If the segmented polygon had only one class reference crowns, the polygon was categorized as “one class merged”. If the segmented polygon had multiple class reference crowns, the polygon was categorized as “multiple class merged”.

(e) Fragmented: If one or multiple reference crowns covered by the segmented polygon, and their respective overlaps were less than 20%, the segmented polygon was considered as a “fragmented polygon”.

We calculated the segmentation accuracy of trees at four areas: Areas 1–4. Area 1 was a deciduous coniferous forest and Area 2 was a strobe pine forest, for which we calculated the entire area. Area 3 was a slash pine and taeda pine forest, for which we calculated part of the areas. Area 4 was a naturally regenerated forest, for which we calculated 1 ha in area. As a result, some segmented images had multiple tree crowns, but this method almost succeeded in separating each tree class (Table 1).

**Table 1:** Accuracy statistics of the tree crown maps. (Area 1: deciduous coniferous tree; Area 2: strobe pine forest; Area 3: slash pine and taeda pine forest; Area 4: naturally regenerated forest).

Forest area	Matched	Nearly matched	Split	Merged		Fragmented
				One class	Multiple class	
Area 1	7	7	13	31	2	17
Area 2	1	0	5	41	2	17
Area 3	19	6	7	27	2	7
Area 4	38	11	23	48	24	16

### Ground truth label attachment to tree crown map

**Figure 3:**
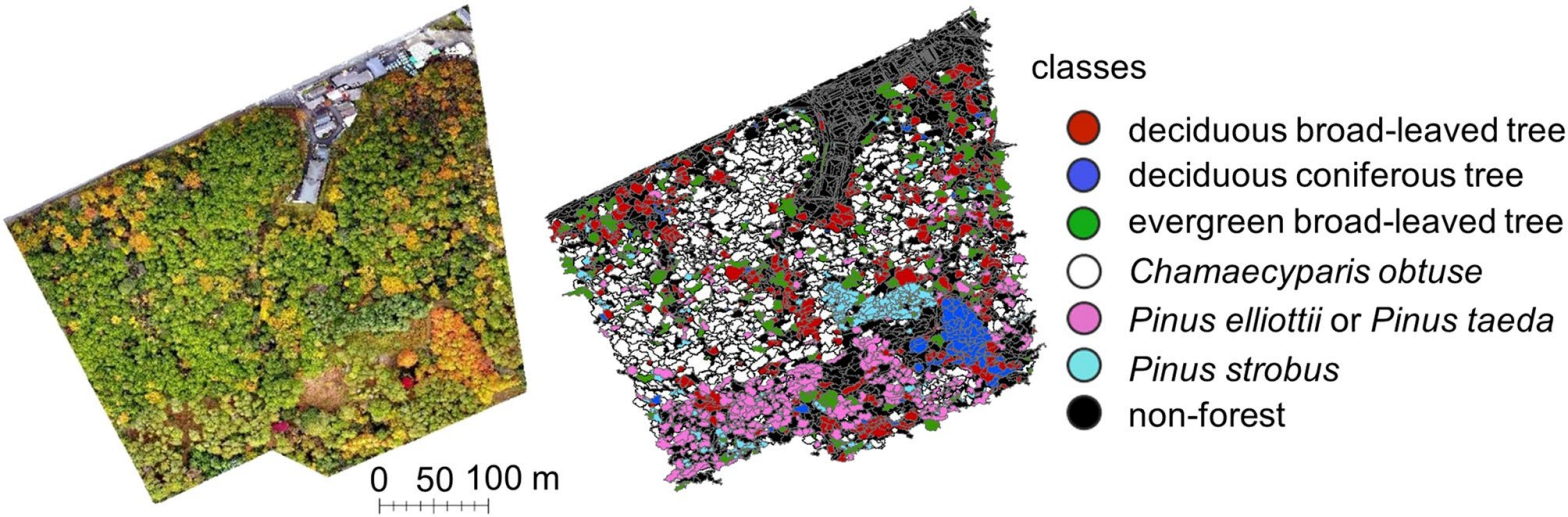
Segmentation and ground truth map-making result. The tree classes found in the image on the left are represented by the colours explained in the legend in the figure on the right. This figure was created using ArcGIS Desktop v10.6 software (https://www.esri.com, Environmental Systems Research Institute, Inc., Redlands, United States).

After segmentation, we classified segmented images into the following seven classes: deciduous broad-leaved tree, deciduous coniferous tree, evergreen broad-leaved tree, *Chamaecyparis* *obtuse*, *Pinus* *elliottii* or *Pinus* *taeda*, *Pinus* *strobus,* and non-forest. The ‘non-forest’ class included understory vegetation and bare land, as well as artificial structures. For deciding these classes, we conducted field research. We set three rectangular plots sized 30 m × 30 m and checked the tree species, regarding the classes we decided could be identified from the November 20 drone images. The *Pinus* *elliottii* or *Pinus* *taeda* class consisted of two *Pinus* species, because these two species are difficult to identify from drone images*.* At the ground truth map-making phase, we visually attached the class label to each tree crown, using nearest neighbour classification in the eCognition software to improve operational efficiency, which was then used for forest mapping^38^ (Fig. 3). More specifically, we chose some image objects as training samples and applied that algorithm to the overall tree crowns. In subsequent steps, by adding wrongly classified objects to correct classes of the training samples, we improved the accuracy of the ground truth map.

### Tree image extraction with ground truth label

**Figure 4:**
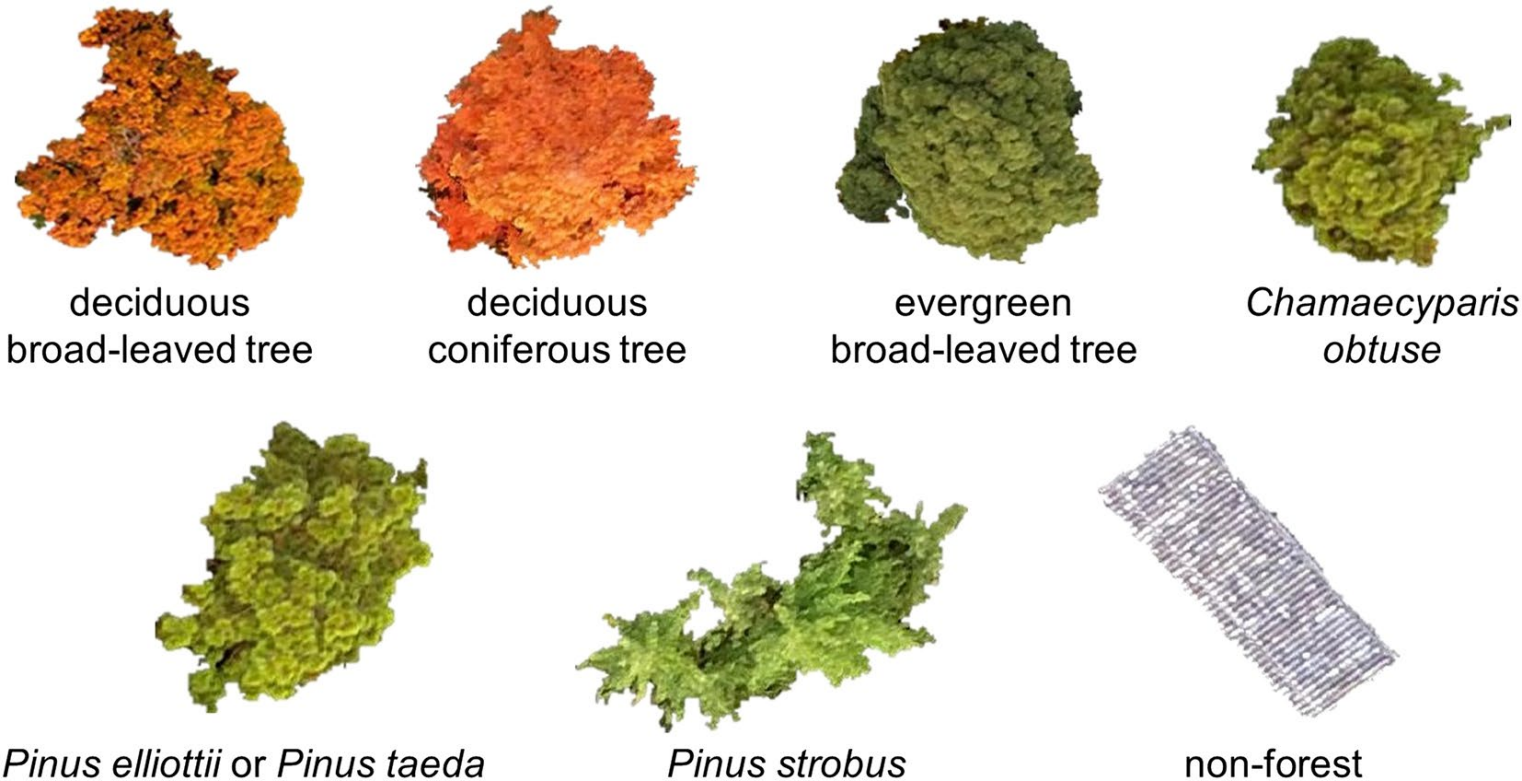
Representative extracted images from each class in the November 20 images. These images were segmented well at each tree crown level. However, the image of *Pinus* *strobus* includes several tree images. The image of the non-forest class shows the roof of a house.

**Figure 5:**
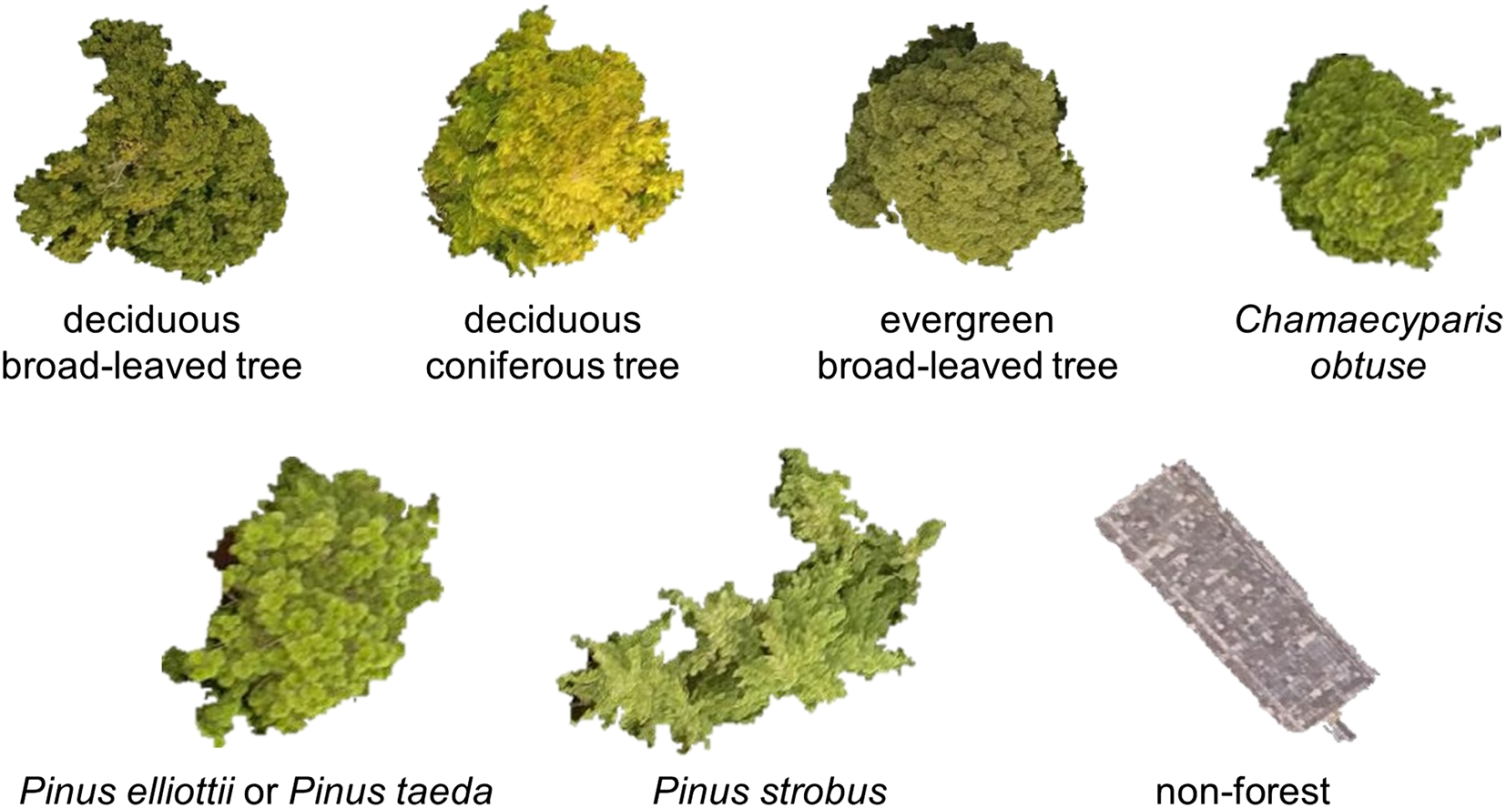
Representative extracted images from each class in the October 2 images. These images were extracted from the same tree crown map polygon as the November 20 images.

From the orthomosaic photos of the two season and the ground truth map, we extracted each tree image with a class label using the ‘Extract by Mask’ function in ArcGIS. There were some inappropriate images, such as fragments of trees, those difficult to be interpreted or classified visually, and those including multiple classes; thus, we manually deleted inappropriate images and placed wrongly classified images into the correct class by group consensus. Representative images of the tree classes are shown in Figs. 4 and 5. The number of extracted images and that of arranged images are shown in Supplementary Table S3. After arrangement, the number of each class ranged from 37 to 416. The images had a wide range of sizes, but the length of one side of the largest image was approximately 400 pixels.

After extraction, we resized the images from October 2 to the size of images from November 20 in order to align the two season conditions. Thus, all images were adjusted to the size of images taken from a height of approximately 100 m.

### Machine learning

To construct a model for object identification, we used the publicly available package PyTorch v0.4.1^39^ as a deep learning framework and four standard neural network models—specifically, AlexNet^23^, VGG16^40^, Resnet18, and Resnet152^41^—for fine-tuning. Fine-tuning is an effective method to improve the learning performance, especially when the amount of data is insufficient for training^42^. We used each neural network model, which had been learned with the ImageNet dataset^43^, and trained all neural network layers using our data. At the CNN training phase, we augmented the training images eight times by flipping and rotating them. Further augmentation did not improve accuracy. For the input to the CNN, we applied ‘random resized crop’ at a scale of 224 × 224 pixel size for training, which crops the given image to a random size and aspect ratio. For validation and training, we resized the images into 256 × 256 pixel sizes and used ‘centre crop’ at a scale of 224 × 224 pixel size. These cropping algorithms extracted only one resized image (patch) from each cropped image. The ranges of the other learning settings are outlined in Supplementary Table S4.

To evaluate the performance of the CNN, we used SVM as a machine learning platform. We used the average and standard deviation of each band and GLCM texture values as features. GLCM is a spatial co-occurrence matrix that computes the relationships of pixel values, and uses these relationships to compute the texture statistics^44^. For calculating GLCM, images with a large number of data bits result in huge computational complexity. In this case, the images that were converted to grey scale were 8-bit data. It is known that reduction of bit size causes only minor decrease in classification accuracy; hence, we rescaled from 8-bit to 5-bit^45,46^. After calculation of GLCM, we extracted five GLCM texture features (angular second moment (ASM), contrast, dissimilarity, entropy, and homogeneity). Their algorithms are defined in Eqs. (1)–(5):

1 $$\begin{array}{c}ASM=\sum_{i,j}^{N}{(P}_{i,j}{)}^{2} \end{array}$$

2 $$\begin{array}{c}Contrast=\sum_{i,j}^{N}{P}_{i,j}{\left(i-j\right)}^{2}\end{array}$$

3 $$\begin{array}{c}Dissimilarity=\sum_{i,j}^{N}{P}_{i,j}\left|i-j\right|\end{array}$$

4 $$\begin{array}{c}Entropy=\sum_{i,j}^{N}{P}_{i,j}\mathrm{log}\left({P}_{i,j}\right)\end{array}$$

5 $$\begin{array}{c}Homogeneity=\sum_{i,j}^{N}{P}_{i,j}/(1+{\left(i-j\right)}^{2})\end{array}$$

where ${P}_{i,j}$ is the GLCM at the pixel which is located in row number *i* and column number *j*. We obtained these GLCM texture features at each pixel, excluding pixels close to the image margin, and then calculated their mean and standard deviation for each image. Another important parameter that affects classification performance is the kernel size^47,48^. To determine the most suitable kernel size for GLCM operation, we calculated GLCM texture features with various kernel sizes of 3, 11, 19, 27, 35, 43, 51, and 59. For SVM validation, we used radial basis function (rbf) kernel and conducted a parameter grid search in the range of gamma from ${10}^{-1}$ to ${10}^{-5}$ and cost from 1 to ${10}^{5}$. As a result of the grid search, we obtained the best validation accuracy and the best parameters at each GLCM kernel size (Supplementary Figure S3). The validation accuracy slightly increased along with the increase in kernel size, and the accuracy stopped increasing at the 51 × 51 kernel size. Considering this result, we adopted the 51 × 51 kernel size and the best parameters as follows: gamma and cost were ${10}^{-2}$ and ${10}^{3}$ in the fall peak season, and ${10}^{-3}$ and ${10}^{4}$ in the green leaf season, respectively. We then used these parameters for SVM learning and the comparative evaluation.

For machine learning, we divided the data into training, validation, and testing sets. The validation dataset was used for hyperparameters tuning such as learning rate, batch size for deep learning, and kernel size, cost, and gamma values for SVM. In the testing phase, we used the data which had not been used for training and parameter tuning. Validation accuracy is not suitable for comparing performance as a final result because validation accuracy can be higher than testing accuracy; we tuned the hyperparameters to get higher accuracy using the validation data. Using testing data, we can exclude the bias of parameter tuning. We also used a kind of cross-validation because we had a limited amount of data and decreased the contingency of accuracy. In this case, we randomly divided all the images evenly into four datasets and used two of them for training, one for validation, and one for testing. Subsequently, we interchanged successively the datasets used for training, validation, and testing. This process was repeated four times. For the accuracy evaluation and confusion matrix, we used total accuracy and all the images.

For this calculation, we used a built to order (BTO) desktop computer with a Xeon E5-2640 CPU, 32 GB RAM, and a Geforce GTX 1080 graphics card; the OS was Ubuntu 16.04.

### Evaluation

For evaluation, we used the overall accuracy, Cohen’s Kappa coefficient^49^, and the macro average F1 score. F1 score is the harmonic mean of Recall and Precision. In this study, the number of images acquired for each class varied significantly. The overall accuracy, which is typically utilised for evaluating the machine learning performance, is subject to the difference in the amount of data available to each class. Therefore, we used the Kappa and F1 score, which is suitable for evaluating imbalanced dataset accuracy, as well as overall accuracy to obtain an objective evaluation. Additionally, for evaluating the per-class accuracy, we used the F1 score of each class.

## Results

### Fall peak season

First, we made four standard neural network models and trained them on the data of the November 20 images. Although Resnet152 exhibited the highest performance, there was little difference among the accuracies of the neural network models (Supplementary Table S5). We therefore adopted Resnet152 as a representative CNN model and denoted it as CNN Model 1.

**Figure 6:**
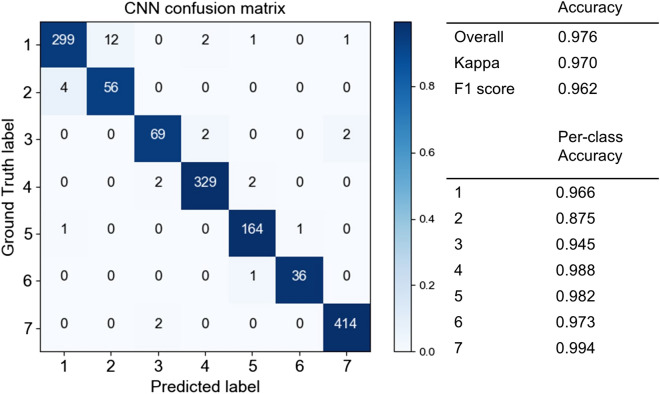
Confusion matrix of CNN in the fall season (Model 1). The vertical axis is the ground truth and the horizontal axis the model prediction. The number in each cell indicates the number of classified images; each cell is coloured according to the percentage of the number of images in each class. (Class 1: deciduous broad-leaved tree; 2: deciduous coniferous tree; 3: evergreen broad-leaved tree; 4: *Chamaecyparis* *obtuse*; 5: *Pinus* *elliottii* or *Pinus* *taeda*; 6: *Pinus* *strobus*; 7: non-forest).

The classification results of CNN Model 1 are shown in Fig. 6; the overall accuracy was 0.976, Kappa 0.970, and F1 score 0.962. Model 1 succeeded in identifying almost all classes with more than 90% accuracy; one class identified with less than 90% accuracy was still identified at a relatively high accuracy greater than 85.0%. The confusion matrix shows the detailed results of model prediction. The vertical axis is the ground truth that we identified visually; the horizontal axis is the class that the model predicted. The number in each cell represents the number of classified images; each cell is coloured by the ratio of number of images per ground truth class. For example, a ratio of 0.0 (light blue) indicates that no image was classified to that cell, whereas a ratio of 1.0 (dark blue) indicates that all images of the ground truth were classified to that cell. From this confusion matrix, it can be seen that almost all classes were classified correctly.

**Figure 7:**
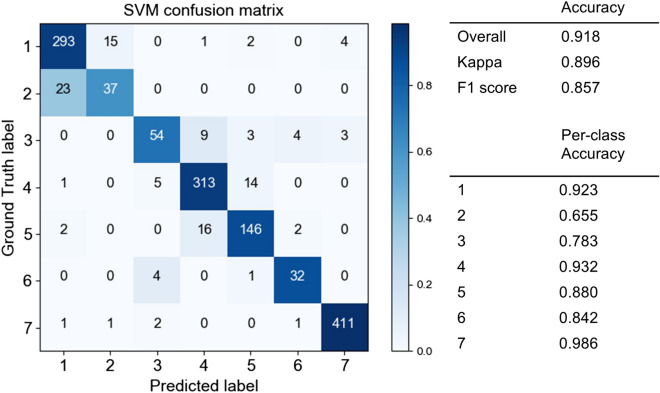
Confusion matrix of SVM in the fall season. (Class 1: deciduous broad-leaved tree; 2: deciduous coniferous tree; 3: evergreen broad-leaved tree; 4: *Chamaecyparis* *obtuse*; 5: *Pinus* *elliottii* or *Pinus* *taeda*; 6: *Pinus* *strobus*; 7: non-forest).

We also tested the SVM method with the same images. The accuracy of the SVM was found to be lower than that of the CNN; however, it also exhibited a high performance (Fig. 7). For the SVM, the overall accuracy, Kappa, and F1 score were 0.918, 0.896, and 0.857, respectively. These values are lower than the corresponding results of the CNN by approximately 6–10%; however, they are still relatively high. Most per-class accuracies are lower than those of the CNN results, especially Class 2, deciduous coniferous tree, which was difficult to identify. As can be seen in the comparison of the confusion matrix of the CNN results to that of the SVM results, misclassifications of the deciduous broad-leaved tree and deciduous coniferous tree, in particular, increased in the latter.

### Green leaf season

**Figure 8:**
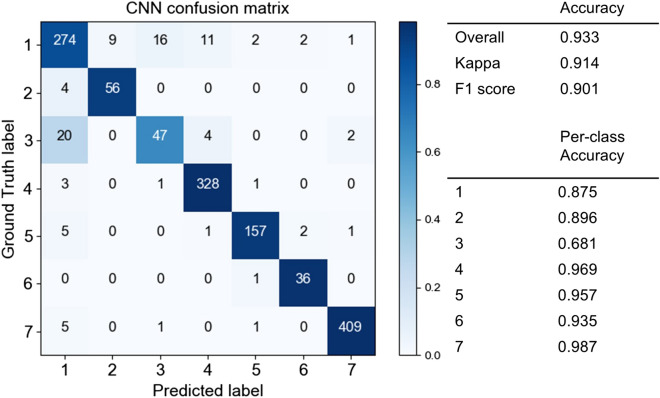
Confusion matrix of the CNN in the leaf season (Model 2). (Class 1: deciduous broad-leaved tree; 2: deciduous coniferous tree; 3: evergreen broad-leaved tree; 4: *Chamaecyparis* *obtuse*; 5: *Pinus* *elliottii* or *Pinus* *taeda*; 6: *Pinus* *strobus*; 7: non-forest).

Next, we constructed four standard neural network models and trained them on the data from the end of the green leaf season. As in the fall peak season, Resnet152 exhibited the highest performance, but the differences in the performance of the models were greater than for that season (Supplementary Table S5). We therefore adopted Resnet152 as a representative CNN model and denoted it as CNN Model 2. We expected that in this season it would be more difficult to identify deciduous classes because the colours of each class are similar. However, Model 2 exhibited significantly higher performance than we expected. The performance of Model 2 is shown in Fig. 8. The overall accuracy, Kappa, and F1 score were 0.933, 0.914, and 0.901, respectively. Per-class accuracies are also high, at approximately 90% in most classes. Compared to misclassifications in the fall season, the misclassification of deciduous broad-leaved trees and evergreen broad-leaved trees in the green leaf season increased.

**Figure 9:**
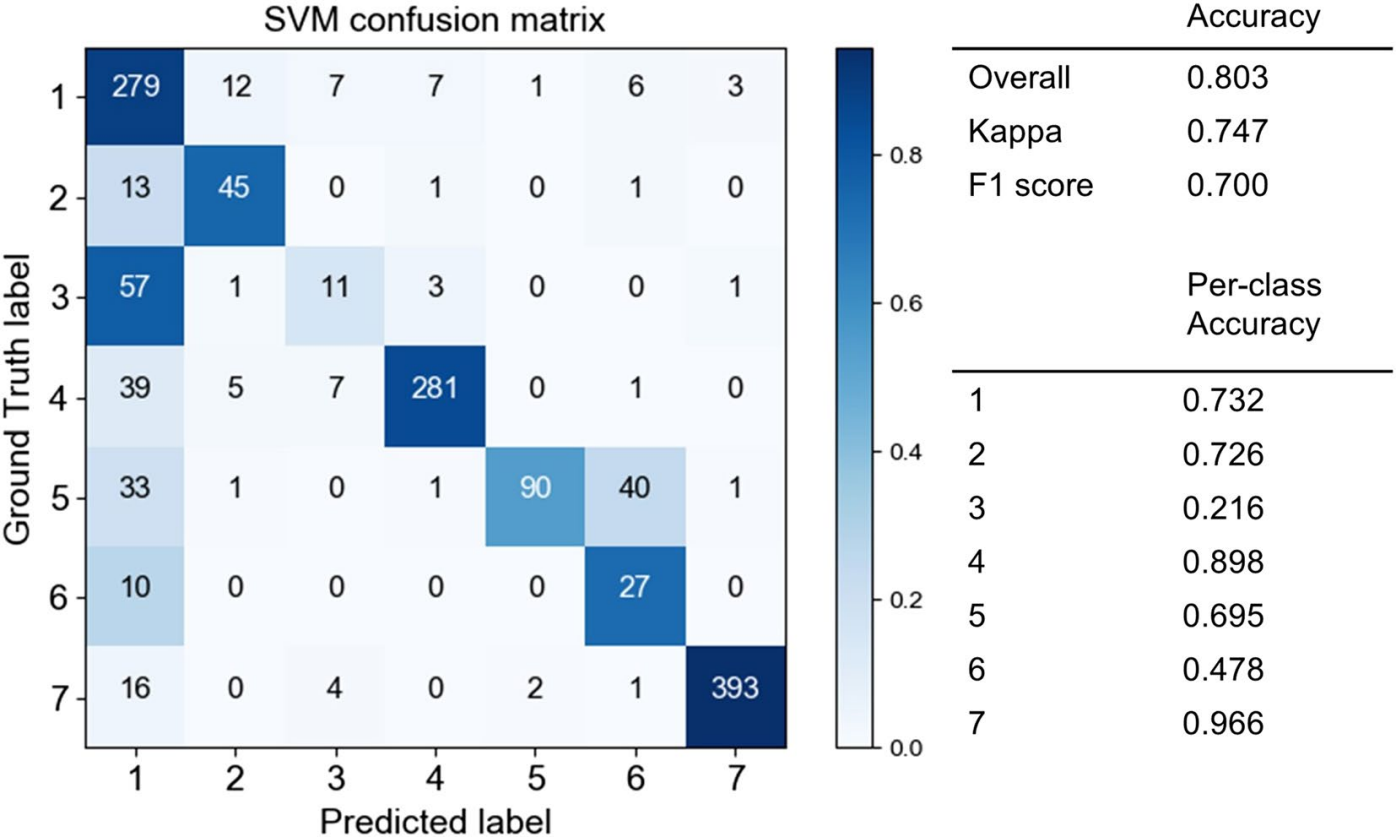
Confusion matrix of SVM in the green leaf season. (Class 1: deciduous broad-leaved tree; 2: deciduous coniferous tree; 3: evergreen broad-leaved tree; 4: *Chamaecyparis* *obtuse*; 5: *Pinus* *elliottii* or *Pinus* *taeda*; 6: *Pinus* *strobus*; 7: non-forest).

We also tested the SVM’s performance with Model 2, wherein the SVM had difficulty identifying these classes (Fig. 9). The overall accuracy decreased to 0.700–0.803, with per-class accuracy of approximately 70–95% for some classes, 48% for the *Pinus* *strobus* class, and 22% for the evergreen broad-leaved tree class. According to the confusion matrix, the evergreen broad-leaved tree class is mostly misclassified as a deciduous broad-leaved tree, whereas the *Pinus* *strobus* is mostly misclassified as the other *Pinus* class and the deciduous broad-leaved tree class.

**Figure 10:**
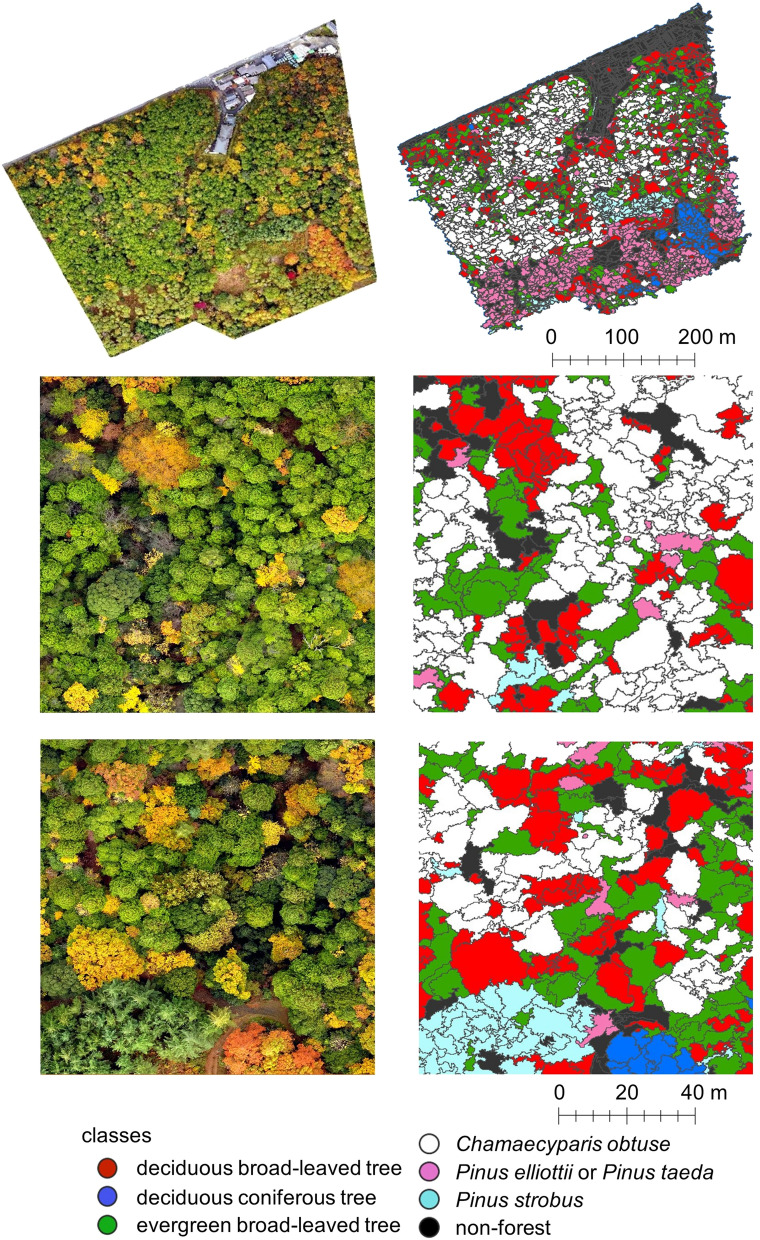
Orthomosaic photos and classification maps obtained with the CNN classifier. The topmost images each gives an overall view of one area, and the lower images in each column show the respective enlarged area. The tree classes found in the images are identified in the legend at the bottom of the figure. This figure was created using ArcGIS Desktop v10.6 software (https://www.esri.com, Environmental Systems Research Institute, Inc., Redlands, United States).

Finally, we visualised the classification results of CNN Model 1 in GIS (Fig. 10). Most of the object images in this figure were used for training; taking this into account, we can see that the CNN succeeded in correctly identifying the trees.

**Figure 11:**
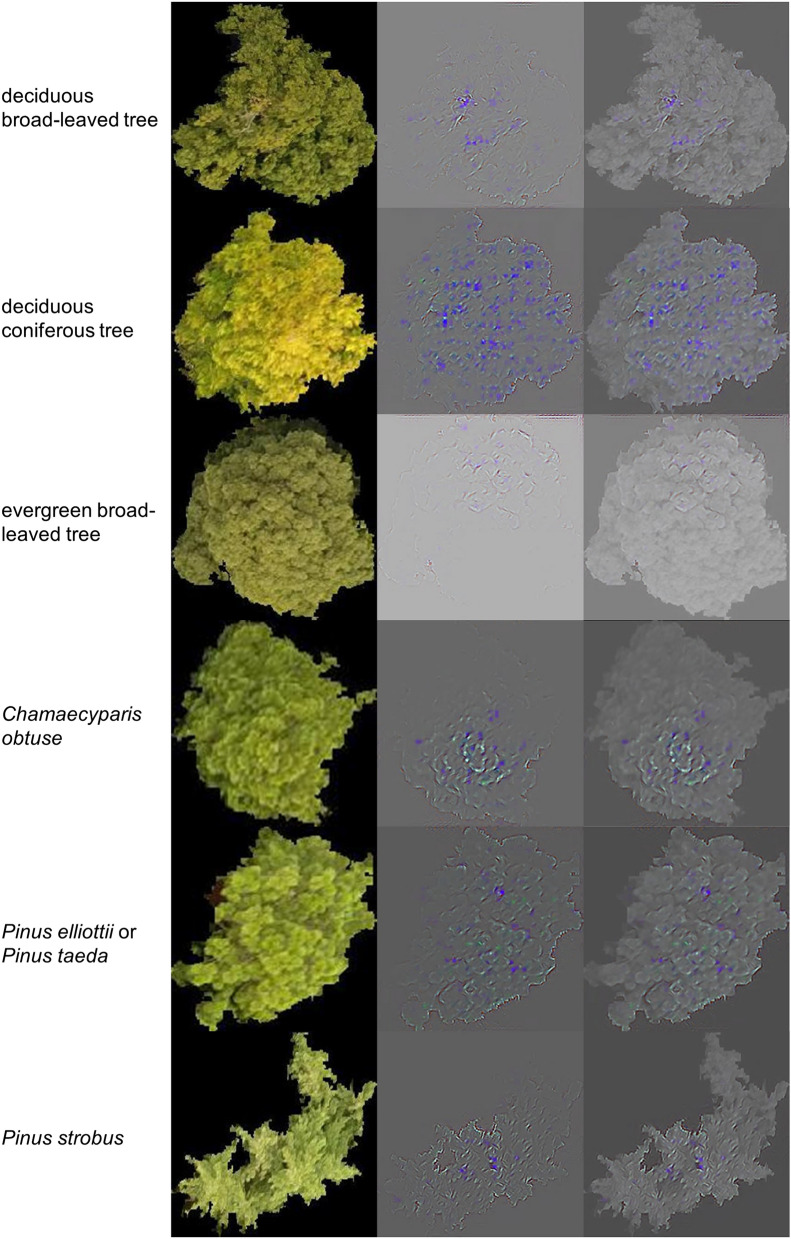
Attention maps of Guided Grad-CAM. (Left to right) class name, original image, result of Guided Grad-CAM, result of Guided Grad-CAM overlaid with the grayscale original image.

Consequently, we applied Guided Grad-CAM for generating an attention map to visualise the features that the CNN used for classification. Guided Grad-CAM can visualise locations and detailed features which are related to the judged class^31^. We applied the algorithms for the classes except for the non-forest class, to layer 4, the last layer of Model 2. Figure 11 shows the original image, the attention map visualised by Guided Grad-CAM, and the grayscale original image overlaid on the highlighted attention map of each class. For deciduous broad-leaved tree, it is difficult to explain clearly what region CNN used. For deciduous coniferous tree, the features are extracted on the whole area, and their locations look to be matched to contrast of edge of the small foliage. For the evergreen broad-leaved tree, though the colour of features is pale, we can see the highlighted features along the edge of the bush of the branch compared to deciduous broad-leaved tree. For *Chamaecyparis* *obtuse,* we can see the features along the edge of the hierarchical branch clearly. For *Pinus* *elliottii* or *Pinus* *taeda* pictures, the features match the edge of the branch. For *Pinus* *strobus*, the outlines of the trees are highlighted.

## Discussion

This study was conducted to investigate the ability of our machine vision system, which combines UAV RGB image and deep learning, to classify individual trees into several tree types and identify specific tree species. Regarding the classification of tree types, Model 1 (pertaining to the fall season) classified four tree types and one non-forest class with Kappa 0.971 and F1 score 0.955, whereas Model 2 classified them with Kappa 0.911 and F1 score 0.882. At the species level, Models 1 and 2 were able to classify *Chamaecyparis* *obtuse*, *Pinus* *strobus* from *Pinus* *elliottii* and *Pinus* *taeda* with more than 90% accuracy. Thus, our system was able to classify tree types and has the potential to classify several tree species in some seasons.

This performance is notable because we used easily available digital RGB images only. Contrastingly, most previous studies used expensive hardware, such as multispectral imagers, to improve performance. Regarding the spatial scale, our method, using a UAV, may be more limited than previous methods using airborne sensors. However, the low-cost and easy-to-use feature of UAVs can enable periodic monitoring. Meanwhile, other researches using UAVs identified only a few tree species or applied rectangle object detection^32–35^. Regarding these points, our system successfully identified several tree classes, and enabled forest mapping. Thus, our machine vision system can be used as a cost-effective and handy tool for application in forest mapping and management.

Comparing the CNN performance to that of the SVM, in the fall peak season, both the SVM and the CNN demonstrated a high performance. However, in the green leaf season, the CNN exhibited a much higher performance than that of the SVM. We used average and standard deviation values of RGB bands and GLCM texture features for SVM learning; thus, this difference in features may have contributed to the performance difference between the SVM and the CNN. This result also indicates that the CNN used more features other than the average and standard deviation values of RGB bands and GLCM texture features.

Applying Guided Grad-CAM, we succeeded in visualising features that CNN used. The results of Guided Grad-CAM suggest that the CNN model used the difference in edge shape of foliage and bush of branch, hierarchical branching pattern and outlines of tree shapes for classification. From these considerations, it is supported that CNN identified trees based on the features of biological structures that represent physiological and ecological characteristics of the target tree classes. This result also indicates that the CNN with UAV image can successfully identify trees according to the differences in structural features, even if trees have similar colours. Considering this fact, high spatial and temporal robustness of this system is expected because if the leaf colour changes owing to factors such as weather or season, it can still be classified correctly using the structural features.

In our method, there are two reasons for the extremely successful result. First, we conducted object-based classification. Previous studies have shown that object-based classification can obtain higher accuracy than pixel-based classification^9,50^. One advantage of applying object segmentation is that each image can have more common features. For example, judging from the image of *Chamaecyparis* *obtuse* in Fig. 11, CNN used the features around the treetop. Using object segmentation, most images can include this feature. However, if we use pixel-based segmentation, each image would be cut by a rectangular image such as 64 × 64 pixel. Therefore, the possibility that the image contains those common features will be low and the accuracy of classification may be lower. Our method could not segment every tree crown perfectly; thus, improving the segmentation method would lead to higher classification accuracy and also enable us to count the number of trees. It is difficult to segment tree crowns using a specific parameter because the tree size and clarity of the treetop are highly diversified, especially in a mixed forest. In future work, applying a deep learning method such as Instance Segmentation^51^ can have high potential for tree crown segmentation.

Second, we selected training and testing images from the same area and the same time. Tree shapes have some variation in different environments, and leaf colours and illuminations are different across periods, weather types, and seasons. Utilisation of tree shapes (or DSM) and the seasonality of leaf colours would improve classification accuracy; however, generally, these properties may have a negative influence on simple machine learning. Considering practicability, a versatile model which involves trained images from various sites and times is desired in further study.

Although we achieved high accuracy classification by using the CNN, we still had some misclassification. Notably, Model 2 struggled to identify deciduous broad-leaved trees and evergreen broad-leaved trees. This misclassification may be attributed to the class inclusion of several tree species, i.e. the branch pattern and colour may not be common across the same tree type. Therefore, separating the classes into more fine-grained branching types or tree species levels may improve the classification accuracy. Thus, the way in which we separated each class may be one of the key parameters affecting classification performance.

Ultimately, we constructed a chain of machine vision systems that can segment and identify trees automatically. Although the classes are limited, this method can be utilised as a base system for tree mapping systems using UAV images.

## Conclusion

In this paper, we proposed a chain of low-cost machine vision system for identifying trees using UAV RGB image and deep learning. Our system achieved an accuracy of more than 90% for classifying tree types and specific tree species. Additionally, the results suggest that the CNN classified trees according to the features of their biological structures such as foliage shapes, and branching pattern; thus, this system may have the potential for identifying several tree species even when the colours of the trees are similar. In a follow-up study, tree crown segmentation using deep learning needs to be conducted, and we are planning to identify more detailed species and also evaluate the spatial and temporal robustness of the developed system.

## Supplementary Information

Supplementary Information.

## Data Availability

The data that support the findings of this study are available from the corresponding author upon reasonable request.
