# Supplementary material for: Explainable identification and mapping of trees using UAV RGB image and deep learning
Source: Sci Rep. 2021 Jan 13;11:903. doi: 10.1038/s41598-020-79653-9 (PMC7806907; doi:10.1038/s41598-020-79653-9)
Supplement: Supplementary file 1 — Supplementary Information. [file 41598_2020_79653_MOESM1_ESM.pdf]

Supplementary Information for:

Explainable identification and mapping of trees using UAV RGB image and deep learning

Masanori Onishi [1,*], Takeshi Ise [2]

[1] Graduate School of Agriculture, Kyoto University, Kyoto, Japan
[2] Field Science Education and Research Centre, Kyoto University, Kyoto, Japan

* Corresponding author: onishi.masanori.25e@st.kyoto-u.ac.jp

This file contains: Supplementary Figures S1-S3 and Tables S1–S5

Supplementary Figures

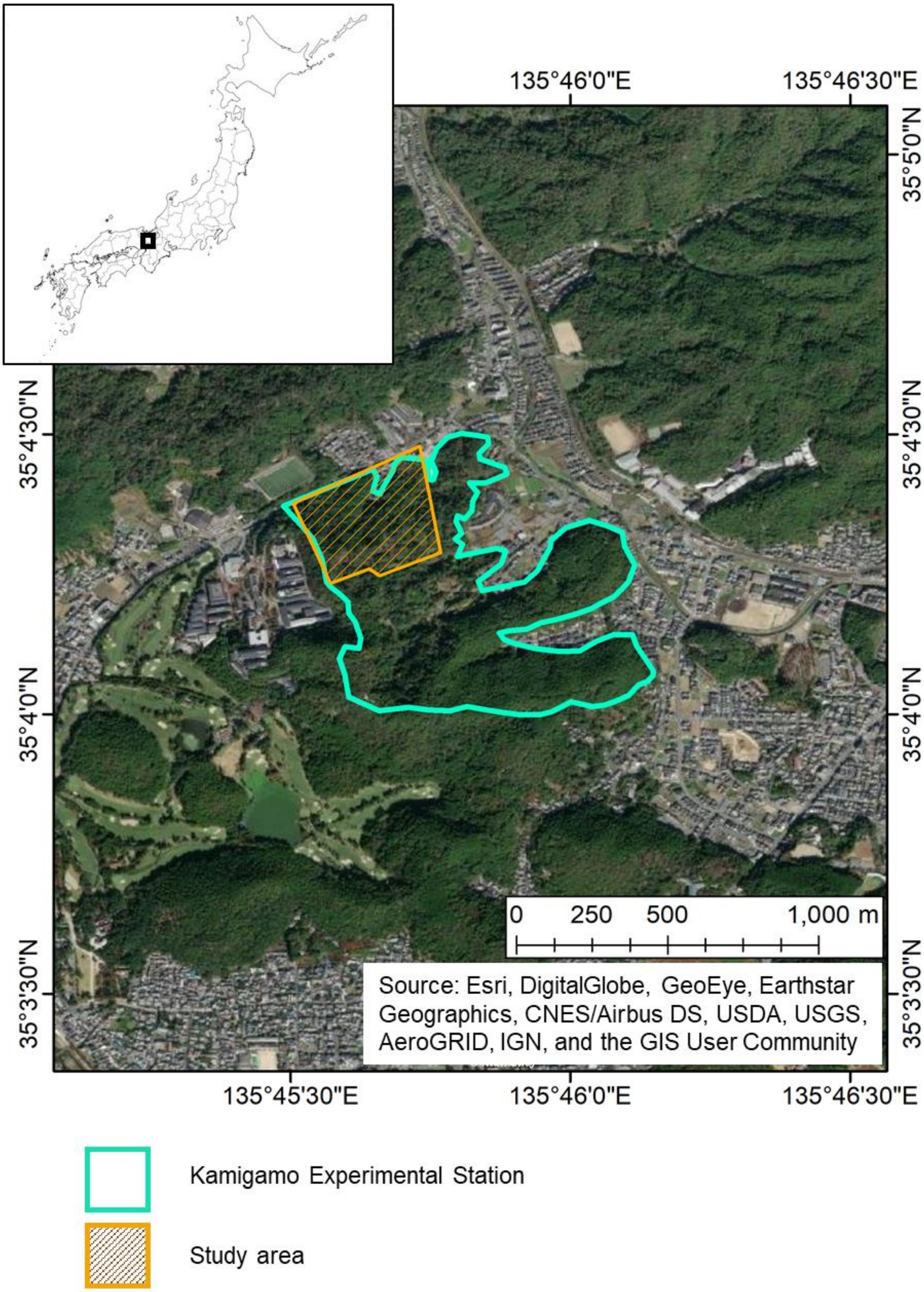

Figure S1. Location map of Kamigamo Experimental Station and study area. This map was created using ArcGIS Desktop v10.6 software (www.esri.com, Environmental Systems Research Institute, Inc., Redlands, United States).

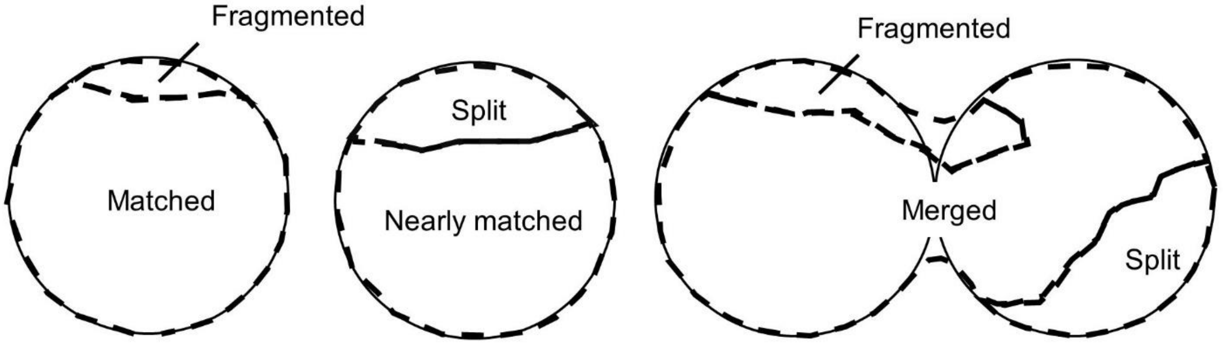

Figure S2. Diagram of the five categories in which segmented tree crowns were placed.

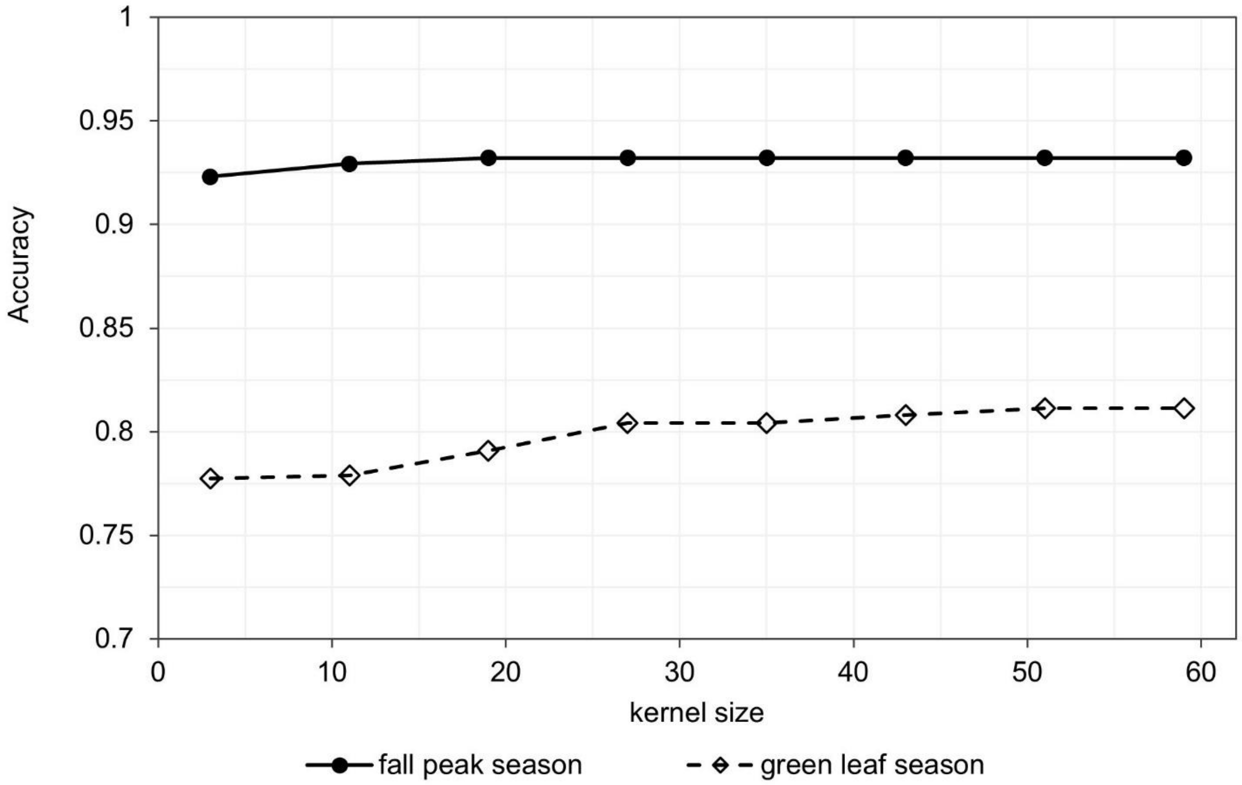

Figure S3. Relationship between GLCM kernel size and SVM validation accuracy in each season.

Supplementary Tables

Table S1. Parameter settings for the workflow used in generating orthomosaic photo.

**Workflow**	**Parameter**	**Settings**
Align Photos Build	Accuracy	Highest
	Preselection	Reference
	Key Point Limit	80,000
	Tie Point Limit	8,000
	Adaptive Camera Model Fitting	Yes
Build Dense Cloud	Quality	High
	Depth Filtering	Aggressive
Build Digital Elevation Model	Projection	JGD2011
	Source	Dense
	Data	Cloud
	Interpolation	Enabled
	Resolution	0.093 m
Build Orthomosaic	Surface	DEM
	Enable Hole Filling	Yes
	Resolution	0.047 m

Table S2. Parameters for multiresolution segmentation.

**Setting**	**Selected option**
Weight of R, G, B, DSM, Slope	1, 1, 1, 2, 3
Scale	200
Compactness	0.5
Shape	0.2

Table S3. Number of images in each class. (These are the values for one dataset).

Class	Extracted	Arranged	Training	Augmented training	Validation	Test
1	418	315	174	1392	70	71
2	87	60	33	264	11	16
3	218	73	28	224	24	21
4	758	333	184	1472	65	84
5	276	166	94	752	38	34
6	129	37	18	144	6	13
7	1439	416	207	1656	110	99

Table S4. Settings of the PyTorch image classification model.

Setting	Selected option
Training epochs	50–100
Batch size	16–64
Solver type	SGD
Base learning rate	0.1–0.001
Momentum	0.90

Table S5. Classification results of neural networks in fall peak season and green leaf season.

**CNN model**	**Fall peak season**			**Green leaf season**		
	Overall	Kappa	F1 score	Overall	Kappa	F1 score
AlexNet	0.960	0.949	0.930	0.902	0.875	0.836
VGG 16	0.973	0.965	0.958	0.925	0.903	0.878
Resnet 18	0.972	0.964	0.954	0.919	0.897	0.876
Resnet 152	0.976	0.970	0.962	0.933	0.914	0.901
